# A Dual-Functional Intelligent Felt-like Label from Cationic Rice Straw Fibers Loaded with Alizarin Red S for Monitoring Al(III) and the Freshness of Fish

**DOI:** 10.3390/foods14162914

**Published:** 2025-08-21

**Authors:** Huiyan Feng, Yikun Li, Qian Cheng, Zhiming Liu

**Affiliations:** Key Laboratory of Bio-Based Materials Science and Technology of Ministry of Education, Northeast Forestry University, No. 26 Hexing Road, Xiangfang District, Harbin 150040, China; 15663526791@163.com (H.F.); sixcharacter00@gmail.com (Y.L.)

**Keywords:** felt-like intelligent label, visual sensing, pH-sensitivity, fish freshness, Al^3+^ detection

## Abstract

To achieve dual functionality that can monitor both Al^3+^ levels in food and the freshness of fish, rice straw fibers (RSFs) were treated in NaOH solutions and then cationized with 2,3-epoxypropyltrimethylammonium chloride, onto which alizarin red S molecules were immobilized through electrostatic interaction to develop a smart felt-like label. An optimized treatment in 5 wt% NaOH solution effectively removed lignin and hemicellulose, facilitating quaternary ammonium group grafting and stable ARS anchoring. The ARS@BRSF-5NaOH exhibited high pH sensitivity, showing visually discernible color changes (ΔE > 5, perceptible to the naked eye) under acidic (pH ≤ 6) and strongly alkaline (pH > 12) conditions. During the storage of the fish, the label transformed from yellow to dark purple (ΔE increase) as TVB-N levels approached 20 mg/100 g, enabling real-time freshness monitoring for protein-rich products. Additionally, the label achieved a detection threshold of 1 × 10^−5^ mol·L^−1^ for Al^3+^ through a coordination-induced chromatic transition (purple to pale pink). This research highlights the feasibility of utilizing an agricultural waste-derived material to develop cost-effective, visually responsive, dual-functional intelligent labels for food safety, offering significant advancements in on-site quality assessment.

## 1. Introduction

In China, Kal(SO_4_)_2_ is a food additive widely used in some foods specifically as a swelling agent and stabilizer for a better texture, such as the chewy texture of vermicelli and the crispiness of deep-fried dough sticks. According to Chinese GB 2760-2014, the maximum allowable amount of aluminum residue in foods is 100 mg/kg [1]. However, excessive use usually occurs to obtain better effects, which can cause serious harm to human beings because Al^3+^ can destroy the nervous system and lead to neurodegenerative diseases such as Alzheimer’s disease and Parkinson’s disease, as well as affect hematopoietic function and interfere with calcium absorption [2,3]. Moreover, the market is flooded with a vast array of food products, making it difficult for ordinary consumers to determine whether aluminum additives exceed safe levels [4,5]. Additionally, protein-rich foods are easily spoiled, releasing organic amines that are dangerous to health. Some seriously spoiled foods must be destroyed, leading to food waste and economic losses. The freshness or spoilage degree of protein-rich foods is often reflected by the total volatile basic nitrogen (TVB-N) content. According to Chinese GB 2733-2015, the TVB-N content of meat and fish must not exceed 15 mg/100 g and 20 mg/100 g, respectively [6]. Generally, people usually judge whether food is spoiled by changes in its color or smell. However, this method may lead to situations where the spoilage is far beyond the standard, and people often cannot detect when food is close to the threshold value in the standard. Until now, the dominant methods for testing aluminum ions in foods or the freshness of protein-rich foods have involved complex operations, required professional personnel, and utilized expensive instruments [7]. For example, according to the Chinese regulation outlined in GB 5009.268-2025, ICP-MS and ICP-OES/AES are used to test Al^3+^ content in standard methods [8]. Consequently, there is a critical need to develop a monitoring method with high sensitivity, low price, and easy operation.

The utilization of intelligent labels that can change colors visually may become a promising approach for daily and real-time monitoring of the quality and safety of foods. A green sensor based on anthocyanin-loaded bacterial cellulose nanofibers achieved the visual detection of aluminum ions (Al^3+^) through color changes. However, this sensor responded to various metal ions, such as Cu^2+^ and Cr^3+^, and the color differences were not significant, resulting in a limited selective recognition for Al^3+^ and reducing the feasibility of practical application [9]. Recently, natural fiber-based sensors have been paid more and more attention due to their precise detection in complex environments. Notably, a colorimetric label combining red cabbage anthocyanin, gelatin, and gallic acid for the visual monitoring of TVB-N in meat was studied [10]. However, when TVB-N concentration was in the range of 20–30 mg/100 g, which was close to or slightly above the national standard limit, the color change gradient of the label was insufficient, making it difficult to distinguish through the naked eye or simple equipment, resulting in a decrease in the matching accuracy between the indication result and the actual degree of spoilage. The limitation indicates the need for intelligent labels with obvious color changes to meet the needs of complex practical applications. To date, no study has been conducted on a dual-functional intelligent label that can specifically recognize aluminum ions (Al^3+^) and respond sensitively to the TVB-N content of protein-rich foods as their freshness changes.

Alizarin red S (ARS) is an anthraquinone derivative and also an acid-base indicator [11,12]. Due to the ortho-diphenolic hydroxyl group, it can chelate Al^3+^ to produce color changes, which originated from Azizi Khereshki’s research [13], and can also respond to acid-base changes due to its different structures and colors at different pH values, which demonstrate its potential for monitoring TVB-N [14,15,16,17]. Although ARS is a dual-functional indicator that can exhibit an obvious color change when in contact with Al^3+^ or TVB-N, it is not edible. So, the fixation is so important to ensure the safety of the intelligent label, which will lead to no potential risk to the monitored protein-rich foods. Therefore, an ideal supporting material is needed. Rice straw is a by-product of rice production, with an annual global output of 650 to 975 million tons [18]. Cellulose fibers in it can be used as a matrix for intelligent labels to open up the possibility of converting agricultural waste into high-value products. Unfortunately, rice straw fibers (RSFs) can physically adsorb only a small amount of intelligent dyes, which are easy to desorb and may cause pollution in the monitored foods. It was proved that cationic modification could make RSFs carry positive charges and adsorb anionic dyes through electrostatic interactions [19]. Epichlorohydrin and triethylamine were used to cationize RSFs in alkaline conditions and then fix bromothymol blue (BTB) and bromocresol purple (BCP) in a NaOH solution [20,21]. However, lipid-soluble epichlorohydrin reacted with cellulose in a biphasic system, and the utilization rate was lower. Therefore, a monophasic modification in which 2,3-epoxypropyltrimethylammonium chloride (EPTAC) is used to cationize cellulose will be more effective [22,23].

Herein, an intelligent label sensitive to both TVB-N and Al^3+^ was fabricated by anchoring ARS onto EPTAC-cationized RSFs through electrovalent bonds. NaOH and alkaline H_2_O_2_ treatments improved the accessibility of RSFs and eliminated the interference of the original color. The morphology of the fibers was observed by scanning electron microscopy (SEM), and the functional groups and micro-structures were characterized by Fourier transform infrared spectroscopy (FTIR), nuclear magnetic resonance spectroscopy (NMR), X-ray photoelectron spectroscopy (XPS), and X-ray diffraction (XRD). In addition, in order to evaluate the application potential of intelligent cellulose-based labels in the actual monitoring of food quality, the response sensitivity of the labels to Al^3+^, pH, ammonia gas, and TVB-N in fish was systematically investigated.

## 2. Materials and Methods

### 2.1. Materials

Raw material (rice straw) was collected from Wuchang, Heilongjiang, China, from which the fibers were separated with an alkali dosage of 15% (using Na_2_O as the calculation basis), a solid–liquid ratio of 1:4, and a total duration of 2 h, including 30 min of holding time and 90 min of heating-up time, achieving a maximum temperature of 165 °C. Hydrogen peroxide (H_2_O_2_, 30 wt%) and sodium hydroxide (NaOH, AR) were obtained from Zhiyuan Chemical Reagent Co., Ltd. (Tianjin, China). 2,3-Epoxypropyltrimethylammonium chloride (EPTAC, AR) was obtained from Keyuan Biochemical Co., Ltd. (Laizhou, China). Ethylenediaminetetraacetic acid (EDTA, AR) was obtained from Tianli Chemical Reagents Ltd. (Tianjin, China). Fresh carp were obtained from a local market.

### 2.2. Preparation of Intelligent Labels

#### 2.2.1. Fabrication of Cationic Rice Straw Fibers

Absolute dry fibers (10 g) were immersed in 100 mL of bleaching solution (NaOH: 5 wt%; H_2_O_2_: 2.5 mL; Na_2_SiO_3_: 1.2 wt%; MgSO_4_: 0.5 wt%; EDTA: 0.5 wt%) at 80 °C for 120 min. Subsequently, the bleached fibers were washed with deionized water repeatedly, and then anhydrous ethanol was used to replace the water to loosen the fibers. Finally, the fibers were filtered and dried, and the bleached rice straw fibers (BRSFs) were obtained.

BRSFs (1.0 g) and 100 mL of the different concentrations of NaOH solution (5, 10, 15, and 20 wt%) were mixed and stirred at ambient temperature for 0.5 h. Afterward, the fibers were washed in deionized water repeatedly, then immersed in anhydrous ethanol to fabricate BRSF-xNaOH (x = 5, 10, 15, and 20). Afterward, 22.59 g EPTAC was added and stirred for 30 min at 65 °C, washed with deionized water, and then soaked with anhydrous ethanol solution. Finally, the EPTAC grafted BRSF-xNaOH (BRSF-xNaOH-Q) was obtained after filtrating and drying.

#### 2.2.2. Adsorption of Alizarin Red S

RBSF-xNaOH-Q (60 mg) was added to 50 mL of a 50 mg/L ARS solution prepared by using 1 g/L NaOH solution as the solvent. The mixture was shaken in a water bath at 30 °C with a shaking speed of 120 rpm for 12 h for the adsorption. After that, the fibers loaded with ARS were washed with distilled water, and the residue solution was diluted to 150 mL. Subsequently, the absorbance of the solution was measured, and the concentration of the ARS solution after the adsorption was calculated using the standard curve (A = 0.02719C). Finally, the adsorption capacity was calculated using the appropriate formula:

(1) $$Q(mg/g)=(50C_{0}-150C_{f})/60$$

where C_0_ and C_f_ are the concentrations of the ARS solution before and after the adsorption, respectively.

#### 2.2.3. Preparation of Intelligent Felt-like Labels

ARS solution (50 mg/L) was obtained by using 1 g/L NaOH as the solvent, to which 20 mg of BRSF-xNaOH-Q was added, and shaken at 30 °C for 12 h. Finally, the fibers were filtered and washed using deionized water and dried to obtain ARS-loaded BRSF-xNaOH-Q, named ARS@BRSF-xNaOH-Q.

ARS@BRSF-xNaOH-Q (60 mg) was used to obtain felt-like intelligent labels by vacuum-assisted filtration. Through the same process, RSF, BRSF, BRSF-xNaOH, and BRSF-xNaOH-Q were also used to prepare felt-like labels for comparisons.

### 2.3. Characterizations

The observation of microscopic morphology of RSF, BRSF-xNaOH-Q, and ARS@BRSF-5NaOH-Q was conducted using a scanning electron microscope (SEM, QUANTA 200, Philips-FEI Co., Hillsboro, OR, USA). The gold-sprayed samples were magnified at a differential voltage of 5 kV. The crystallinity was tested with an X-ray diffractometer (XRD, XRD-6100 Shimadzu, Kyoto, Japan). The elemental composition and functional groups were determined separately by X–ray photoelectron spectroscopy using an AXIS Ultra DLD (XPS, Ultra DLD, Manchester, UK) and Fourier transform infrared spectroscopy (FTIR, Nicolet iS50, ThermoFisher, Waltham, MA, USA), from 500 to 4000 cm^−1^ with a resolution of 4 cm^−1^. The structure change after grafting quaternized groups was demonstrated by using a nuclear magnetic resonance spectrometer (NMR, Bruker Avance Neo 400WB, Berlin, BB, Germany).

### 2.4. Response Experiments

#### 2.4.1. pH Response

To determine the pH-responsiveness of the felt-like labels, they were cut into discs with a diameter of 0.6 cm and soaked in 5 mL of different pH-buffer solutions (pH 2~13), until their colors completely changed. After the felts were dried, the color was recorded, and the chromaticity parameters—L* (Lightness), A* (Green-Red Axis), and B* (Blue-Yellow Axis)—were tested with a handheld colorimeter. ΔE was obtained using the following formula:

(2) $$∆E\;=\;\sqrt{(A*-A_{i}*{)}^{2}+(B*-B_{i}*{)}^{2}+(L*-L_{i}*{)}^{2}}$$

where L_i_*, A_i_*, and B_i_* are the chromaticity parameters of the initial label, and are denoted by L*, A*, and B* after response, respectively.

#### 2.4.2. Al^3+^ Response

All samples were cut into pieces with a diameter of 0.6 cm for the response experiments. Al(NO_3_)_3_ ethanol solutions with different concentrations (10^−1^, 10^−2^, 10^−3^, 10^−4^, 10^−5^, 10^−6^ mol/L) were prepared. The ARS@BRSF-xNaOH-Qs (x = 5, 10, 15, 20) were separately placed into 10 mL, 30 mL, and 50 mL of Al(NO_3_)_3_ solutions, respectively, and the color of the labels was observed and recorded after 2 h. The label treated with different concentrations of alkali was placed into 10 mL of Al(NO_3_)_3_ ethanol solution at a concentration of 10^−1^ mol/L and taken out at regular intervals (1 min) to record the color parameters.

#### 2.4.3. NH_3_ Response

Circular felt-like labels with a diameter of 0.6 cm were placed in a buffer solution with a pH of 3. After the color of the labels turned yellow, they were washed with distilled water and then dried. Subsequently, they were, respectively, placed in jars with different humidity levels of 11%, 33%, 53%, and 75% and kept for 24 h, then taken out and, respectively, pasted onto the top of a transparent plastic box. Then, 0.01 mL of ammonium hydroxide (25%) was immediately injected into the box (450 mL), and the color changes of the labels after different time intervals were recorded.

#### 2.4.4. Pollution Evaluation and TVB-N Response

ARS@BRSF-5NaOH-Q (d = 1.5 cm) was wrapped in white tissue paper, which easily absorbs water, then exposed to two humidity levels (43% and 75%) for 24 h. Then, the pollution was examined by tissue paper discoloration.

A very fresh fish (carp) was processed to obtain skin-peeled pieces (approximately 1.5 cm × 1.5 cm). Fish pieces (400 g) were accurately weighed and placed in a petri dish with ARS@BRSF-5NaOH-Q (d = 0.6 cm). The color of the label at regular intervals was observed and recorded to assess any color changes during storage. After observing the color, the TVB-N of the fish sample was measured according to Chinese standard GB5009.228-2016 [24].

### 2.5. Statistical Analysis

All results were performed in triplicate and conducted using variance (ANOVA) with Duncan’s multiple-range tests in SPSS Statistics 25.0. Significant differences between groups were indicated with different letters (a–k).

## 3. Results and Discussion

### 3.1. Characterization of the BRSF and BRSF-xNaOH

#### 3.1.1. Morphologies of RSF, BRSF, and BRSF-xNaOH

**Figure 1:**
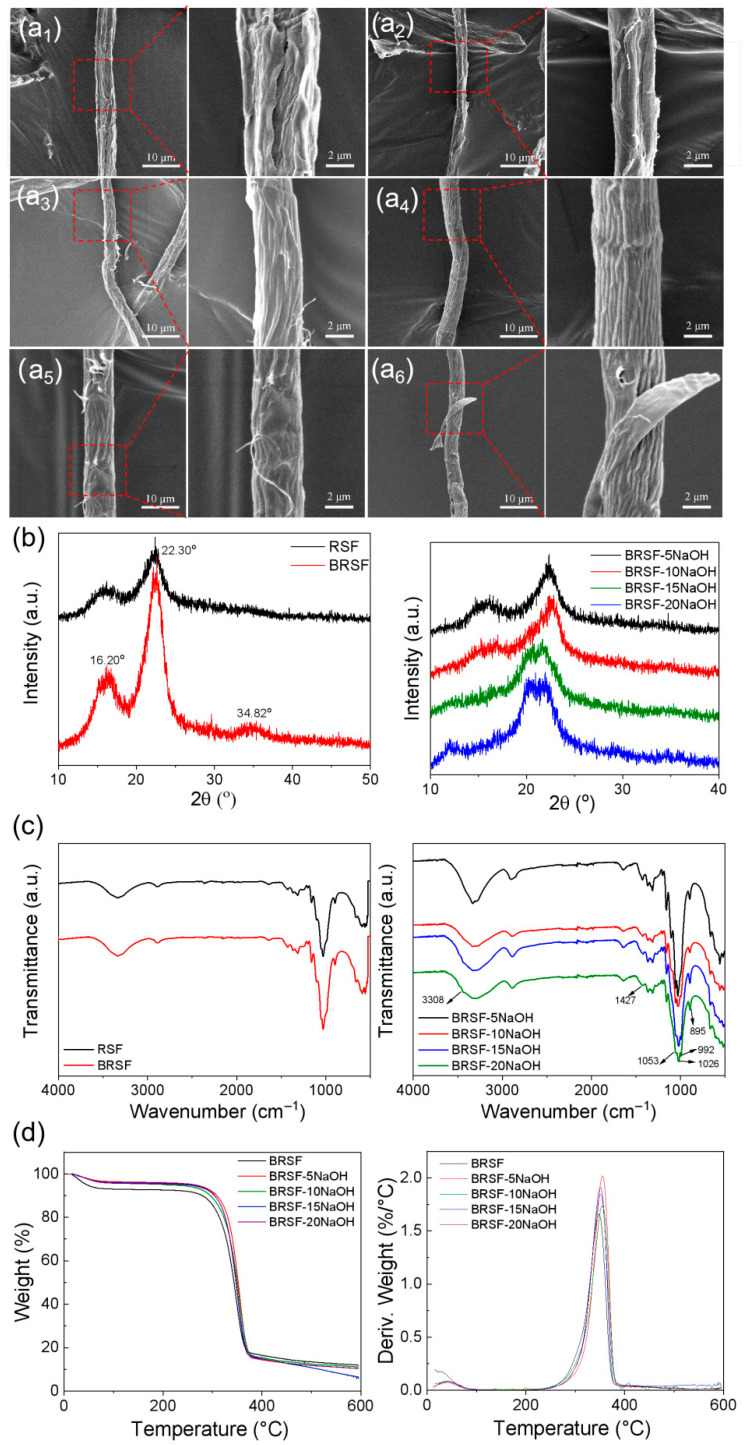
SEM images of (**a1**) RSF, (**a2**) BRSF, (**a3**) BRSF-5NaOH, (**a4**) BRSF-10NaOH, (**a5**) BRSF-15NaOH, (**a6**) BRSF-20NaOH; XRD, (**b**) FTIR, (**c**), and thermal stability (**d**) of the samples.

As shown in Figure 1(a1–a6), there were a lot of longitudinal grooves on the surface of the rice straw fiber (RSF) because most of the lignin and part of the hemicellulose in the cell wall were removed in the separating process of the fibers from rice straw, making the surface become wrinkled and wizen. After bleaching, as seen in Figure 1(a2), the surface was still wrinkled and the grooves were deepened, with some fine “hairs” occurring. This resulted from the removal of lignin and hemicellulose and the packaged cellulose fibrils on the surface separating from the fiber. Additionally, Figure 1(a3–a6) shows that the grooves became more serried and more fibrils upwarped with the increase in the concentration of NaOH from 5% to 15%, which was associated with more significant removal of the lignin and hemicellulose surrounding the fibrils. When the concentration of NaOH reached 20%, cracks appeared, and a slice was peeled off from the bulk of BRSF. As a result of this, treatment in an excessively high concentration of alkali might not only remove hemicellulose but also lead to a slight degradation of cellulose molecules.

#### 3.1.2. Structures of RSF, BRSF, and BRSF-xNaOH

Figure 1b presents the XRD patterns of RSF, BRSF, and BRSF-xNaOH. RSFs exhibited characteristic peaks at 2θ = 16.20°, 22.30°, and 34.82°, representing the crystal planes (101), (002), and (040) of the typical cellulose I structure [25]. After bleaching, the peak at 22.30° became sharper due to the increase in crystallinity, which was attributed to the elimination of the amorphous regions caused by hemicellulose and lignin in RSF. Compared with BRSF, the peaks at 16.20° of BRSF-xNaOH became broader and weaker because NaOH disrupted the ordering arrangement of cellulose chains, resulting in a decrease in crystallinity. It is worth noting that in XRD patterns of BRSF-15NaOH and BRSF-20NaOH, new double peaks appeared at approximately 2θ = 22.0°, indicating a partial structural lattice transformation from cellulose I to cellulose II due to high-concentration NaOH [26]. This could be explained by the fact that sodium hydroxide broke the intermolecular hydrogen bonds and weakened the hydrophobic interactions of cellulose [27], facilitating the penetration of NaOH solution into the crystals, leading to the rearrangement of the internal structure of cellulose and the formation of a more stable cellulose II.

As shown in Figure 1c, for RSF, the peaks at 3308 cm^−1^ and 2892 cm^−1^ originated from the stretching vibrations of O-H and C-H, respectively. The peak at 1637 cm^−1^ was due to the C = O stretching vibration of the conjugated carbonyl group in lignin. The peaks at 1423 cm^−1^ and 1236 cm^−1^ were attributed to the C-H bending vibration in the aromatic skeleton and the stretching vibration of the benzene ring-O bond in lignin, respectively. The peaks at 1053 cm^−1^ and 1026 cm^−1^ were attributed to the C-O stretching in cellulose and hemicellulose. The peaks at 992 cm^−1^ and 895 cm^−1^ were caused by the stretching vibrations of C-O-C in the pyranose ring skeleton and the β-glycosidic bond in cellulose, respectively [28]. After bleaching, the O-H peak at 3300–3500 cm^−1^ showed reduced intensity and narrowed width, indicating hydrogen bonding networks were disrupted and the free hydroxyl groups increased. Subtle modifications in the C-H region at 2800~3000 cm^−1^ suggested altered chemical environments. Upon treatment with 5~20% NaOH solution, increasing alkali concentration led to progressive intensity decline of the O-H peak at 3300~3500 cm^−1^ due to further cleavage of hydrogen bonds. The diminishing peaks at 1053 cm^−1^ (C-O) and 895 cm^−1^ (β-glycosidic bond) reflected altered bond environments and partial structural damage. Concurrently, reduced intensity at 1423 cm^−1^ (aromatic CH_2_) and 1236 cm^−1^ (benzene-O) demonstrated NaOH-induced modifications in lignin’s aromatic skeleton conformation and electron cloud redistribution. These changes comprehensively hinted at the impacts of bleaching and alkali-treating on the structures and properties of lignin, cellulose, and hemicellulose in RSFs and BRSFs.

#### 3.1.3. Thermal Stability

Derivative thermogravimetry (DTG) and thermogravimetry (TG) of BRSF and BRSF-xNaOH are illustrated in Figure 1d. The pyrolysis of BRSF was divided into two stages. The first stage was below 109.34 °C, indicating that water molecules evaporated. The second stage, from 242.93 to 391.75 °C, was attributed to the thermal decomposition of hemicellulose and cellulose. During the thermal decomposition, the maximum decomposition temperature occurred at 347.81 °C. After that, the residual carbon content was 11.94%. After alkali treatment, the maximum decomposition temperatures of BRSF-5NaOH, BRSF-10NaOH, BRSF-15NaOH, and BRSF-20NaOH were 359.22 °C, 354.6 °C, 350.39 °C, and 350.86 °C, respectively, which were slightly higher than that of BRSF because of the partial removal of hemicellulose. Additionally, with an increase in alkali treatment concentration, the maximum decomposition temperature of BRSF-xNaOH decreased, as excessive alkali concentration might reduce crystallinity because of the transformation of cellulose crystalline from I to II, resulting in the fibers being pyrolyzed more easily.

### 3.2. Analyses of BRSF-xNaOH-Q

#### 3.2.1. ^13^C NMR Spectra

**Figure 2:**
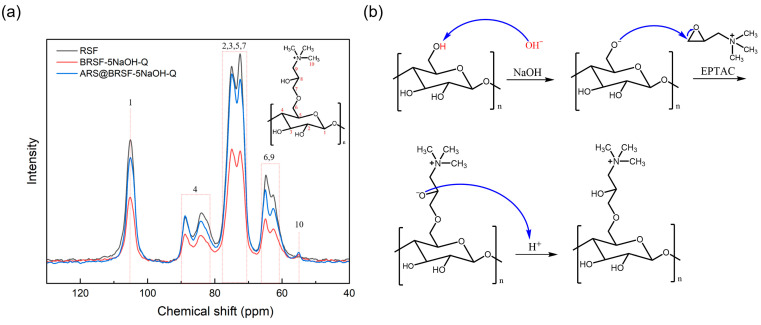
^13^C NMR of RSF, BRSF-5NaOH-Q, and BRSF-5NaOH-Q (**a**) and mechanism scheme of grating process (**b**).

The ^13^C NMR spectra of RSF and BRSF-5NaOH-Q are shown in Figure 2a. Compared with RSF, a new peak marked as 10 occurred at 54.98 ppm, belonging to the quaternary ammonium salt, which indicated that the grafting of EPTAC on BRSF was successful. Additionally, the peak marked as 6 was slightly shifted to a higher field, which implied the grafting reaction happened on –OH groups connected with the carbon atom marked as 6 in the cellulose molecules of BRSF.

The grafting process is shown in Figure 2b. NaOH deprotonated the hydroxyl groups of the cellulose molecules, generating highly nucleophilic oxyanions. In the epoxy ring of EPTAC, the significantly higher electronegativity of the oxygen made the two carbon atoms bonded to the oxygen exhibit electropositivity and have electrophilicity. The oxyanions generated from the cellulose, acting as nucleophiles, attacked the carbon atom in the epoxy ring with stronger electropositivity according to the SN2 nucleophilic substitution mechanism because nucleophiles tend to attack sites with low electron-cloud density and electropositivity. After the attack, the epoxy ring opened, the chloride ion departed, and a quaternary ammonium-containing side-chain was simultaneously introduced. Therefore, the quaternary ammonium salt was successfully grafted onto BRSF-xNaOH.

#### 3.2.2. XPS Analysis

The atomic percentages of C and N were obtained in the XPS survey spectra. Then, through the peak deconvolution of the N1s peak, shown in Figure S1, two peaks near 400 eV and near 402.8 eV were obtained, which corresponded, respectively, to C-N and C-N^+^ [29,30]. The atomic percentage of C-N^+^ could be obtained by the area ratio of the two peaks. Then, the percentage of carbon atoms in EPTAC and the percentage of carbon atoms in the glucose unit were obtained. Thus, the ratio of the number of C-N^+^ to the number of glucose units was obtained. XPS results of BRSF-xNaOH-Q are shown in Table 1. As the concentration of NaOH increased from 5% to 20%, the ratio of C-N+ to glucose decreased, which hinted that the proportion of EPTAC grafting was the highest when the concentration of the alkali was 5%. According to the reacting mechanism, excess OH^−^ anions could compete with cellulose oxygen negative ions to open the epoxy rings; therefore, the increased side reaction made the grafting decrease. Simultaneously, the atomic percentage of C increased relatively.

#### 3.2.3. XRD Analysis

After grafting EPTAC, the XRD pattern of BRSF-5NaOH-Q still clearly retained the typical characteristic peaks of cellulose I at 2θ = 16.0° and 22.6°. This indicated that under the treatment of 5% NaOH, the grafting reaction of EPTAC mainly occurred in the amorphous region or on the crystal surface of cellulose. The phenomenon might be attributed to the limited degree of damage to the hydrogen-bond network in cellulose fibers by the low-concentration alkaline solution, which made it difficult for the quaternary ammonium groups to penetrate into the deep crystalline region, thus avoiding significant changes in the cellulose I crystal structure. However, when NaOH concentration was increased to 10% (BRSF-10NaOH-Q), the epoxy group of EPTAC undergoes a ring-opening reaction with the hydroxyl groups of cellulose. The generated quaternary ammonium groups triggered a structural transformation through a dual effect: on the one hand, the electrostatic repulsion effect of the positively charged -N^+^(CH_3_)_3_ groups weakened the intermolecular hydrogen bonds of cellulose; on the other hand, its steric hindrance effect further hindered the orderly packing of molecular chains. The synergistic effect led to a significant decrease in the intensity of the characteristic peak of cellulose I (2θ = 16.0° and 22.6°) and an increase in the full width at half maximum. At the same time, characteristic peaks of cellulose II appeared at 2θ = 12.2° and 20.3°. When NaOH concentration was further increased to 15~20% (BRSF-15NaOH-Q/BRSF-20NaOH-Q), the strong alkaline environment promoted the deep swelling of cellulose, exposing more active hydroxyl sites. A small amount of quaternary ammonium groups grafted inside cellulose due to the alkali swelling, causing the further formation of cellulose II. The lattice expansion of cellulose caused by the alkali treatment reduced the resistance to chain segment movement, and the continuous insertion of quaternary ammonium groups completely destroyed the original hydrogen-bond network. The dual effects ultimately resulted in the partial disappearance of the characteristic peaks of cellulose I in the XRD spectrum and the formation of dominant peaks of cellulose II at 2θ = 12.2°, 20.3°, and 21.8°.

#### 3.2.4. Morphologies of BRSF-xNaOH-Q

**Figure 3:**
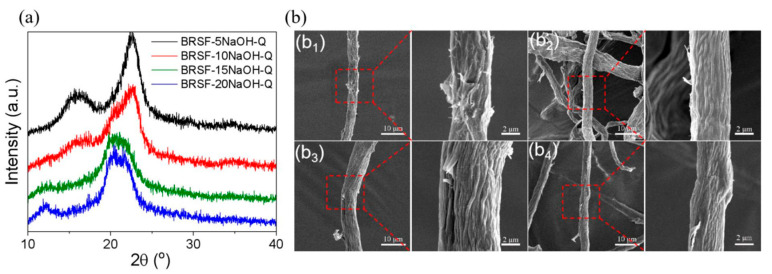
XRD (**a**) and SEM images (**b**) ((**b1**) BRSF-5NaOH-Q; (**b2**) BRSF-10NaOH-Q; (**b3**) BRSF-15NaOH-Q; (**b4**) BRSF-20NaOH-Q) of BRSF-xNaOH-Q.

SEM images of BRSF-xNaOH-Q are shown in Figure 3b. A silk mesh structure was observed on the surface of BRSF-5NaOH-Q and BRSF-10NaOH-Q, probably belonging to quaternary ammonium salts. However, there was nearly no difference in morphology among those BRSF-xNaOH-Q because the wrinkled surface was associated with the removal of lignin and hemicellulose, and also the degradation of cellulose in NaOH treatment.

### 3.3. Characterizations ARS@BRSF-xNaOH-Q

A decrease in ARS adsorption capacity on BRSF-xNaOH-Q from 66.5% to 11.3% was observed with treatment in NaOH concentration from 5% to 20%, as presented in Table 2. The alkali treatment enhanced the accessibility of cellulose, which was beneficial for the grafting of quaternary ammonium groups to adsorb the anionic ARS. However, ARS-loading was also related to the quantity of quaternary ammonium groups due to electrostatic interactions, so the adsorption capacity result was consistent with the XPS analysis mentioned in Table 1. A similar trend was observed in the corresponding color parameters. When NaOH concentration was increased to 10%, the cellulose appeared deep purple, with A* and B* values of 21.30 and −11.58, indicating that ARS imparted red and blue tones to the cationic cellulose fibers. However, as NaOH concentration was further increased, the fibers gradually turned light purple, and the absolute values of A* and B* decreased due to a decline in the adsorption capacity of ARS on the quaternized cellulose.

**Figure 4:**
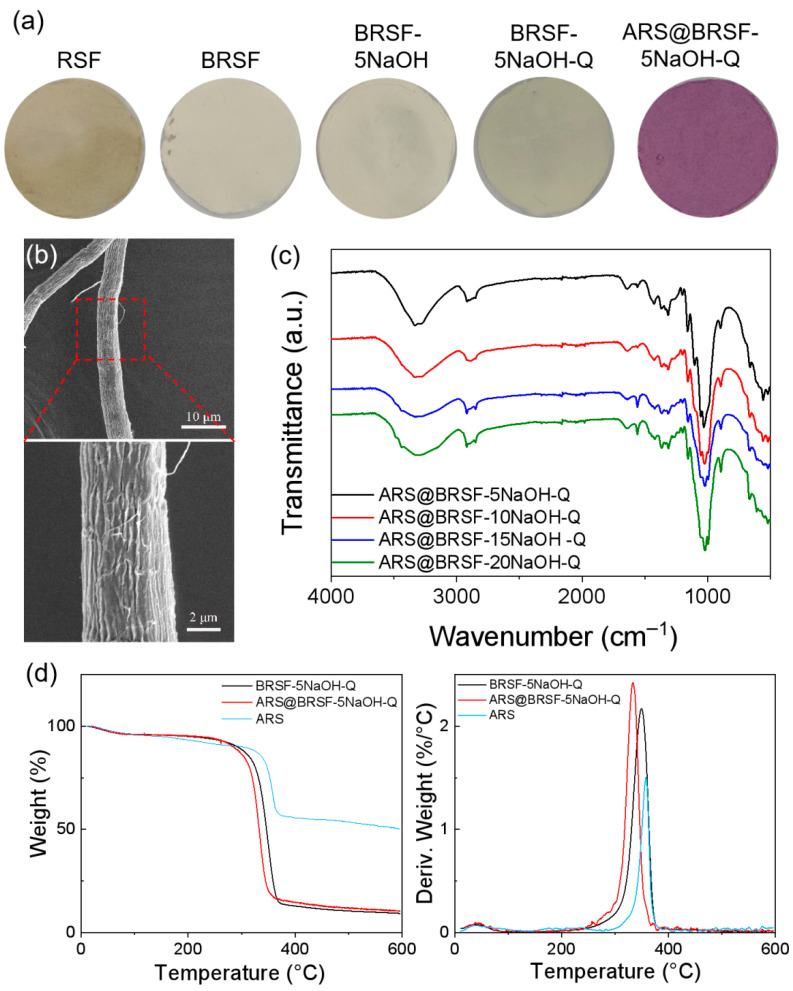
Photos of the samples (**a**), SEM images of ARS@BRSF-5NaOH-Q (**b**), FTIR of ARS@BRSF-xNaOH-Q (**c**), and thermal stability (**d**).

Figure 4a shows the photos of the samples: RSF was yellowish-brown, while BRSF, BRSF-5NaOH, and BRSF-5NaOH-Q were white. After absorbing ARS, the label (ARS@BRSF-5NaOH-Q) was purple. As shown in Figure 4b, the surface morphology of ARS@BRSF-5NaOH-Q was similar to that of ARS@BRSF-xNaOH-Q, probably because the ARS content was so low that the morphology was not affected.

Figure 4c shows FTIR spectra of ARS@BRSF-xNaOH-Q. Compared with BRSF-xNaOH-Q, a new band appeared at 1559 cm^−1^, belonging to the stretching vibrations of the aromatic structure in ARS, indicating that it was successfully loaded on BRSF-xNaOH-Q.

The TG and DTG of ARS, BRSF-5NaOH-Q, and ARS@BRSF-5NaOH-Q are shown in Figure 4d. The maximum decomposition temperatures of ARS, BRSF-5NaOH-Q, and ARS@BRSF-5NaOH-Q were 358.61 °C, 349.20 °C, and 325.22 °C. Compared with BRSF-5NaOH, the maximum decomposition temperature of BRSF-5NaOH-Q was lower because of the lower crystallinity, which was the same reason the maximum decomposition temperature of ARS@BRSF-5NaOH-Q was lower than that of BRSF-5NaOH-Q.

**Figure 5:**
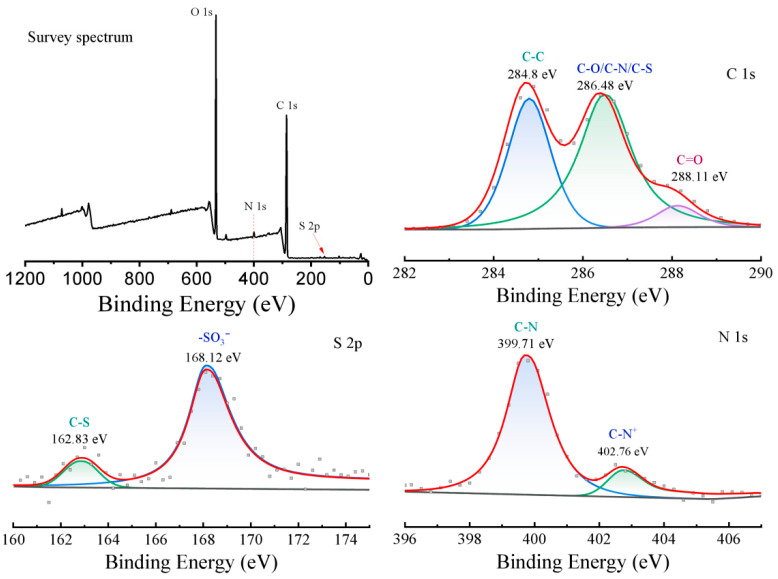
XRS spectra of ARS@BRSF-5NaOH-Q.

As shown in Figure 5, in the XPS survey spectrum, characteristic peaks such as O 1s, C 1s, N 1s, and S 2p are clearly presented, confirming the presence of oxygen, carbon, nitrogen, and sulfur elements in the sample. In the C 1 s spectrum, the peak was meticulously decomposed into multiple sub-peaks. Among them, the binding energy of 284.8 eV resulted from the C-C bond, which was a chemical bond type abundantly present in the rice straw fiber itself. At the binding energy of 286.48 eV, the peaks of C-O, C-S, and C-N overlap, further demonstrating the successful introduction of quaternary ammonium groups and the successful combination with ARS. The binding energy of 288.11 eV represented the existence of C=O. In the S 2 p spectrum, the peak at 168.12 eV corresponded to the sulfonic acid group (-SO_3_^−^) in ARS molecules, and the peak at 162.83 eV represented the C-S bond, confirming the successful loading of ARS onto the fiber surface. The N 1s spectrum was deconvoluted into C-N (399.71 eV) and C-N^+^ (402.76 eV). The binding energy of C-N^+^ was lower than that before the interaction with ARS (402.86 eV) due to the decreasing electron density, which provides strong evidence for the electrostatic interaction between the quaternary ammonium cations and ARS [31].

### 3.4. Response to Al^3+^

The Al^3+^-sensitivity of ARS@BRSF-5NaOH-Q is displayed in Table 3. When the concentration of Al^3+^ increased, the color of the smart labels changed from purple to pale pink due to the formation of complexes from ARS and Al^3+^. Moreover, ΔE was 11.61 when the concentration of Al^3+^ > 10^−5^ mol/L, where there was a low enough concentration for Al^3+^ testing. ΔE was 13.93 at c(Al^3+^) = 10^−4^ mol/L and 27.55 at c(Al^3+^) = 10^−3^ mol/L but a slight decrease occurred to 24.38 at c(Al^3+^) = 10^−2^ mol/L. These results show the relationship between ΔE and concentration, and indicate excellent Al^3+^-sensitivity. It was found that the Al^3+^-detection capability of the intelligent label was closer to the LOQ (limit of quantitation) of Al^3+^ concentration of 0.5 mg/L (1.8 × 10^−5^ mol/L) recorded in Chinese GB 5009.268-2025, proving its practical utilization in daily life [8].

**Figure 6:**
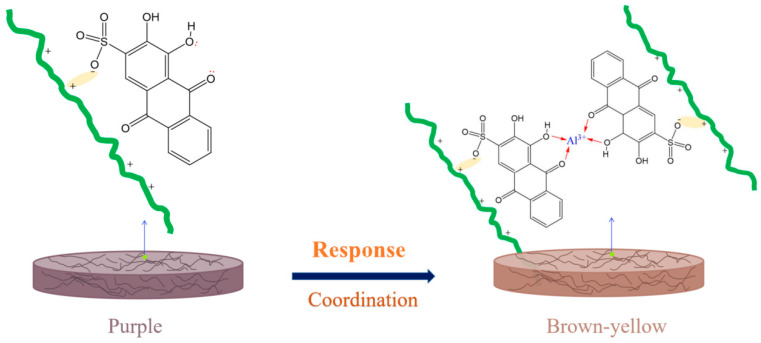
Mechanism schematic of coordination between Al^3+^ and alizarin red S.

As shown in Figure 6, the change was due to the coordination between Al^3+^ and alizarin red S in the smart tags. In the ARS molecule, the O atoms of carbonyl and hydroxyl could provide two electron couples, making it the electron donor and a Lewis base. The outermost electrons of Al^3+^ were empty, which allowed it to form a complex with ARS (Lewis base) in the coordination process. This coordination process achieved a significant color change and made the response between smart tags and Al^3+^ reasonable and reliable.

### 3.5. Response to pH

**Figure 7:**
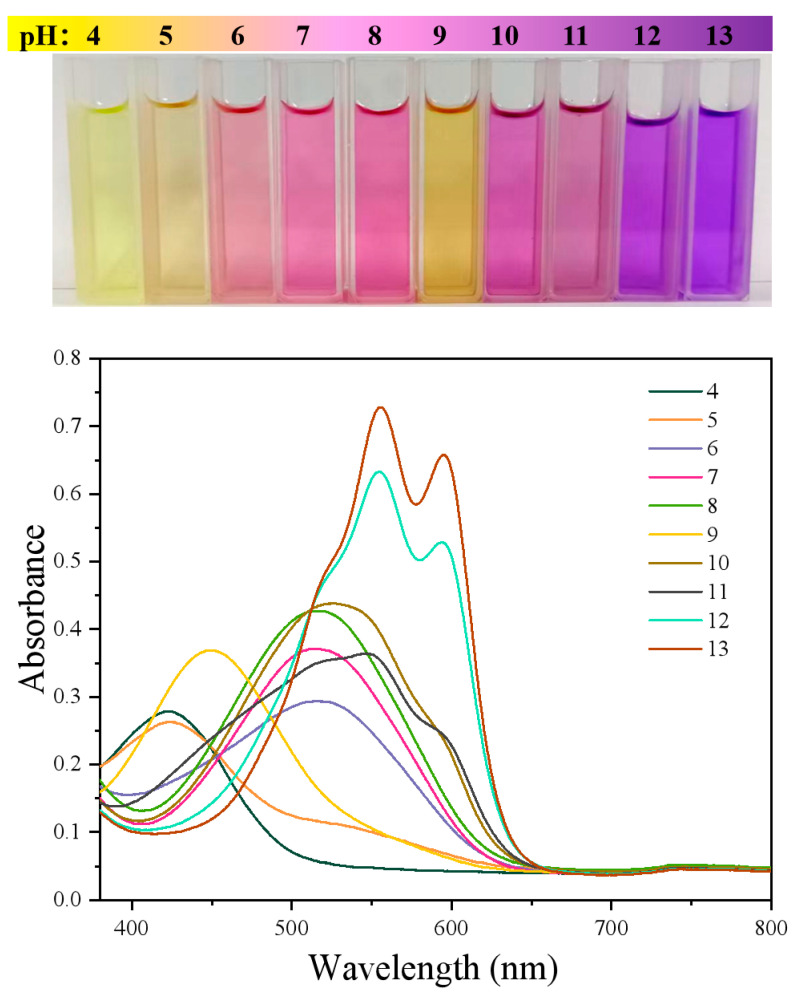
Colors and Uv-vis spectra of ARS at different pHs.

As shown in Figure 7, the visible-light absorption peak was at 423 nm, corresponding to purple light, when the pH was 4 and 5. Therefore, ARS appeared yellow-green. When the pH ranged from 6 to 8, the peak was located at 516 nm, indicating the absorption of green light. ARS appeared purplish-red. When the pH was 9, the peak appeared at 451 nm, which proved the absorption of blue light, and ARS was yellow. When the pH was 10, the peak was at 526 nm, which also corresponded to green light. At that moment, ARS was also purplish-red. When the pH was 11, the three absorption peaks merged into a broad one. Besides the peak at 526 nm, two absorption peaks at 554 nm and 594 nm appeared, indicating the simultaneous absorption of green light and yellow light. Therefore, the color was a mixture of purplish-red and blue. When the pH was further increased to 11 and 12, the peaks at 554 nm and 594 nm dominated. At that time, yellow light was mainly absorbed, so the color tended to be more bluish [32,33].

**Figure 8:**
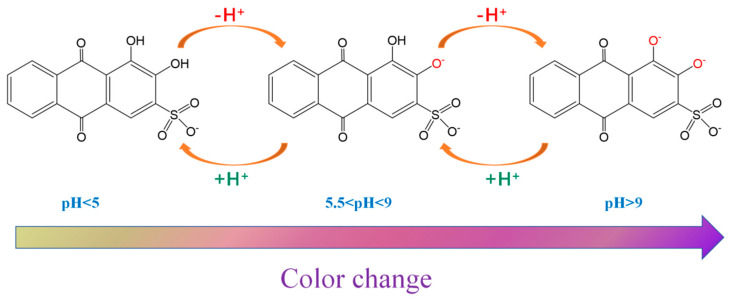
Mechanism of ARS color change in different pHs.

This color-changing phenomenon was attributed to the states of the two phenolic hydroxyl groups of ARS, with the mechanism shown in Figure 8. When pH < 5, ARS had two phenolic hydroxyl groups. Under pH between 5.5 and 9, the phenolic hydroxyl group adjacent to the sulfonate group underwent deprotonation easily due to the electron-withdrawing effect of the sulfonate group [34]. This effect reduced the electron cloud density of the phenolic hydroxyl group, thereby facilitating proton dissociation. In contrast, the phenolic hydroxyl group closer to the carbonyl group tended to form an intramolecular hydrogen bond with the carbonyl oxygen, which stabilized its protonated form and made its deprotonation less favorable compared to the sulfonate-proximal hydroxyl group. At pH > 9, both phenolic hydroxyl groups deprotonated. This pH-dependent color variation was attributed to different deprotonation states of ARS. The pH-sensitivity of ARS@BRSF-5NaOH-Q is shown in Table 4. When the pH was lower than 3, the color of ARS@BRSF-5NaOH-Q was yellow. As the pH was increased from 4 to 8, the color turned light purple. However, the color of ARS@BRSF-5NaOH-Q was gray-brown at pH = 9. Then, when the pH changed from 10 to 13, the color tended to be dark purple. The total lightness decreased in the pH series. The significant color change from yellow to purple appeared as the pH increased from 3, which could be distinguished by the naked human eye. Therefore, acidic ARS@BRSF-5NaOH-Q had a visible sensitivity to OH^−^, providing its potential for monitoring protein-rich products.

### 3.6. Response to NH_3_

**Figure 9:**
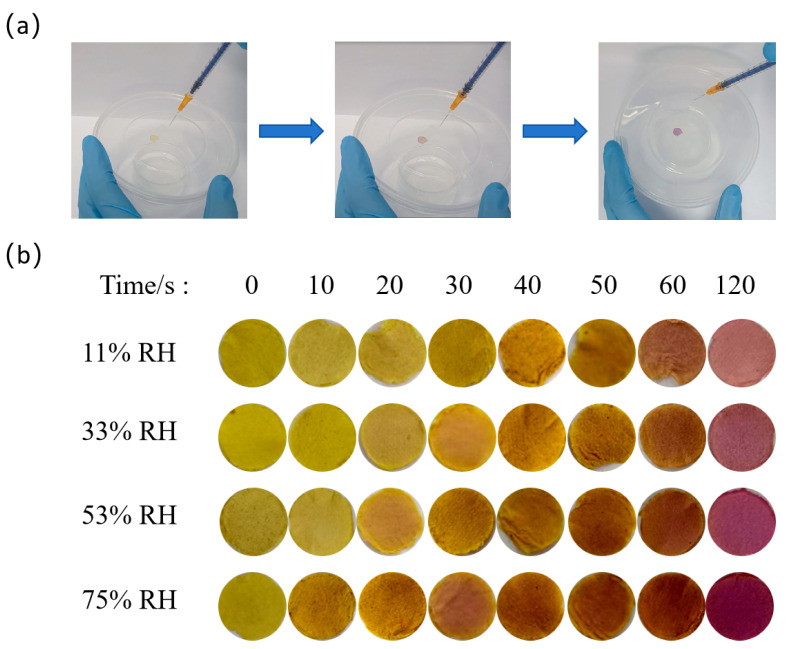
Photos of NH_3_ response experimental process (**a**) and colors of labels with different relative humidity and times (**b**).

As shown in Figure 9, the colors of the labels changed from yellow to purple over time, which demonstrates the visual response process of the labels to NH_3_. When the relative humidity of the label was 11%, the response process took the longest time, approximately 120 s. When the relative humidity of the label reached 75%, the response time was the shortest, approximately 60 s. According to Brønsted–Lowry acid-base theory [35], water molecules acted as an acid (H^+^ donor) and transferred their protons to the nitrogen atom of NH_3_ (H^+^ acceptor), generating OH^−^ and NH_4_^+^, then the label interacted with OH^−^ to visually exhibit the color change. The higher relative humidity resulted in higher water content in the label, which made the process faster.

### 3.7. Pollution Evaluation of ARS@BRSF-5NaOH-Q

**Figure 10:**
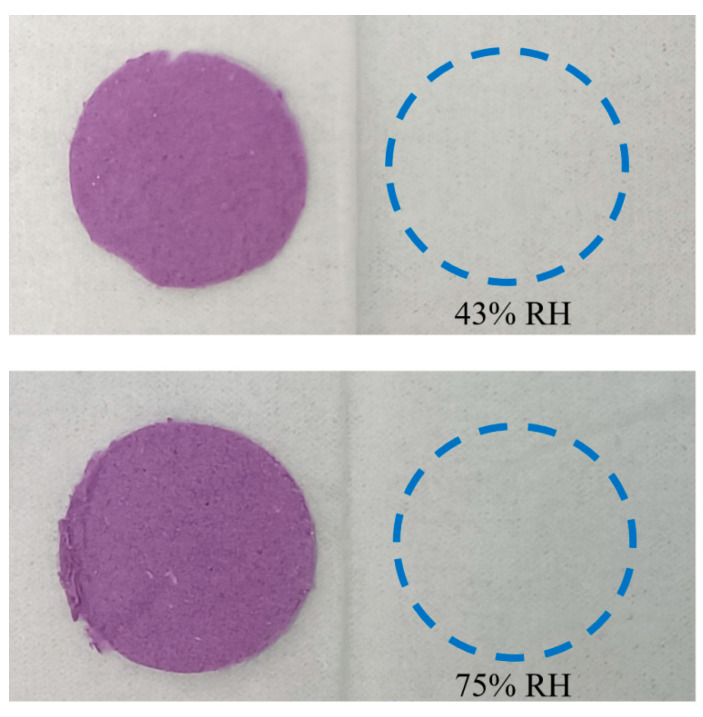
Discoloration of ARS@BRSF-5NaOH-Q observed after 24 h exposure to humidity of 43% and 75%.

As shown in Figure 10, there was no color on the tissue paper in humidity of 43% and 75% after 24 h contact with the label, which further proved that the strong electrostatic interaction between BRSF-5NaOH-Q and ARS existed, and the strategy in this study was successful. Therefore, the label would be used safely because ARS would not migrate to pollute the supervised foods.

### 3.8. Response to TVB-N

**Figure 11:**
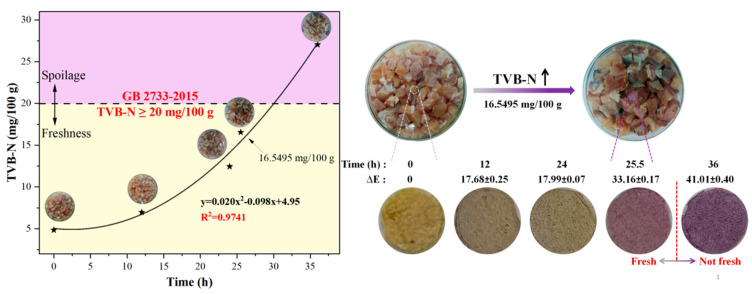
Relationship between TVB-N levels of fish and time, and correlation of color change (ΔE) with TVB-N [6].

**Figure 12:**
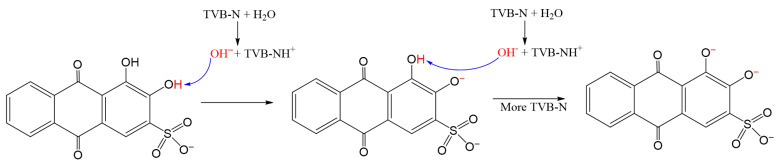
Mechanism of the intelligent felt’s response to TVB-N.

Analyzing TVB-N and the color difference (ΔE) of fish can prove whether the quality of fish can be effectively monitored through a smart label or not. As shown in Figure 11, with the increase in storage time, the TVB-N value significantly increased, and when the TVB-N value exceeded 20 mg/100 g, the fish had entered the spoilage stage. Additionally, Figure 11 shows a significant correlation between times and TVB-N, which could be expressed using the equation y = 0.02x^2^ − 0.098x + 4.95 (R^2^ = 0.9741), reflecting that the fish’s freshness change was a time-dependent process under the experimental conditions. Moreover, the label color changed from yellow to dark purple from 0 h to 36 h, and ΔE increased from 0 to 41.01, which was much higher than values in other studies [36,37]. There was a significant color change from light purple to dark purple as TVB-N approached 20 at 25.5 h. This color change could be explained by the Brønsted–Lowry acid-base theory, according to which TVB-N reacts with H_2_O in the label to generate OH^−^ and TVB-NH^+^. The generated OH^−^ would change the state of the phenolic hydroxyl group. When a small amount of OH^−^ came from lower-concentration TVB-N, a phenolic hydroxyl group converted into a phenolate anion. A larger amount of hydroxide ions caused the same cleavage on both phenolic hydroxyl groups, leading to the color change of the label, as shown in Figure 12.

Those results emphasize the feasibility of smart labels in assessing fish quality, indicating that color-changing labels can provide consumers and producers with intuitive and timely methods for judging the quality status. Additionally, compared with the other studies shown in Table 5, our intelligent felt-like label (0.28 cm^2^) could respond to 400 g of fish, proving its higher efficiency and better feasibility of practical application than those in other studies.

## 4. Conclusions

In this study, a dual-functional intelligent felt-like label based on ARS-loaded cationic cellulose was successfully fabricated for monitoring fish freshness and Al^3+^ levels in food products. FTIR, SEM, XPS, and XRD characterization provided information on chemical structure and morphology changes during the intelligent label’s preparation to confirm the successful adsorption of ARS onto BRSF-5NaOH-Q. The ARS@BRSF-5NaOH-Q label exhibited excellent sensitivity to TVB-N, demonstrating a progressive chromatic shift from yellow (fresh state) to pink (spoiled state) when TVB-N reached 16.5459 mg/100 mg, slightly lower than the threshold value in the Chinese national standard of 20 mg/100 mg. Furthermore, the label was found to have Al^3+^ sensitivity by the color transition from purple to pale pink, providing the quantitative visualization of Al^3+^ at 10^−5^ mol/L level. The dual-functional response can provide some information on the quality of protein-rich foods and Al^3+^ levels in certain foods. In conclusion, this felt-like intelligent label has the potential to become a simple test tool for assessing food quality.

## Figures and Tables

**Table 1:** The XPS analysis results of BRSF-xNaOH-Q.

	C (%)	N (%)	C-N^+^: C-N	C_Glu_ (%)	C-N^+^: C_Glu_
BRSF-5NaOH	30.81	0.62	0.50	28.95	0.39
BRSF-10NaOH	41.01	0.94	0.29	39.40	0.25
BRSF-15NaOH	57.82	0.57	0.31	56.77	0.11
BRSF-20NaOH	58.16	0.44	0.29	57.41	0.08

**Table 2:** Effects of NaOH treatment concentration on the adsorption capacity and color parameters of ARS-loaded BRSF-xNaOH-Q.

	Q (mg/g)	Photo	L*	A*	B*
ARS@BRSF- 5NaOH-Q	66.50 ± 1.42	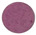	51.68 ± 0.90 ^b^	21.20 ± 0.78 ^a^	−13.04 ± 0.93 ^d^
ARS@BRSF- 10NaOH-Q	41.96 ± 0.45	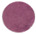	50.18 ± 0.74 ^c^	21.30 ± 0.48 ^a^	−11.58 ± 0.91 ^c^
ARS@BRSF- 15NaOH-Q	12.27 ± 0.03	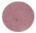	63.96 ± 0.48 ^a^	14.82 ± 0.11 ^b^	−3.36 ± 0.20 ^b^
ARS@BRSF- 20NaOH-Q	11.30 ± 0.13	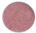	64.08 ± 0.60 ^a^	18.20 ± 0.19 ^c^	−1.74 ± 0.04 ^a^

^a–d^ For each samples, means with different lowercase letters in the same column are significantly different (*p* < 0.05).

**Table 3:** Effect of c(Al^3+^) on color parameters and ΔE on BRSF-5NaOH-Q.

c(Al^3+^) (mol/L)	Photo	L*	A*	B*	ΔE
0	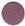	53.65 ± 0.16	18.39 ± 0.08	−9.39 ± 0.10	-
10^−6^	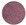	53.46 ± 0.11 ^e^	18.46 ± 0.10 ^e^	−7.59 ± 0.09 ^e^	1.82 ± 0.08 ^e^
10^−5^	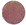	56.29 ± 0.29 ^d^	19.13 ± 0.22 ^d^	1.89 ± 0.32 ^d^	11.61 ± 0.36 ^d^
10^−4^	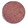	59.08 ± 0.38 ^c^	19.80 ± 0.18 ^c^	3.36 ± 0.44 ^c^	13.93 ± 0.54 ^c^
10^−3^	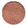	64.74 ± 0.17 ^b^	21.32 ± 0.25 ^b^	15.66 ± 0.34 ^a^	27.55 ± 0.28 ^a^
10^−2^	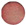	66.56 ± 0.40 ^a^	22.11 ± 0.33 ^a^	10.96 ± 0.07 ^b^	24.38 ± 0.10 ^b^

^a–e^ For each samples, means with different lowercase letters in the same column are significantly different (*p* < 0.05).

**Table 4:** Effect of pH on color parameters and ΔE on ARS@BRSF-5NaOH-Q.

pH	Photo	L*	A*	B*	∆E
Original	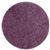	51.83 ± 0.03	17.38 ± 0.02	−9.92 ± 0.08	0
2	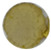	73.44 ± 0.06 ^b^	3.68 ± 0.01 ^h^	23.66 ± 0.10 ^b^	40.10 ± 0.11 ^b^
3	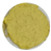	76.04 ± 0.05 ^a^	3.37 ± 0.02 ^h^	31.15 ± 0.07 ^a^	47.65 ± 0.03 ^a^
4	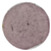	65.97 ± 0.29 ^d^	10.48 ± 0.14 ^g^	−0.60 ± 0.09 ^d^	15.79 ± 0.32 ^d^
5	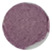	56.91 ± 0.05 ^i^	14.44 ± 0.00 ^c^	−4.21 ± 0.01 ^f^	5.96 ± 0.01 ^i^
6	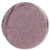	64.22 ± 0.02 ^e^	13.76 ± 0.18 ^d^	−1.79 ± 0.02 ^e^	12.85 ± 0.04 ^e^
7	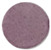	63.60 ± 0.13 ^f^	13.01 ± 0.03 ^e^	−4.18 ± 0.04 ^f^	11.30 ± 0.14 ^f^
8	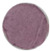	60.96 ± 0.13 ^h^	13.83 ± 0.05 ^d^	−5.09 ± 0.03 ^g^	8.41 ± 0.10 ^g^
9	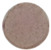	66.65 ± 0.01 ^c^	10.96 ± 0.02 ^f^	3.81 ± 0.05 ^c^	18.84 ± 0.04 ^c^
10	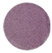	61.04 ± 0.27 ^h^	14.30 ± 0.20 ^c^	−7.39 ± 0.18 ^h^	7.56 ± 0.34 ^h^
11	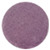	62.11 ± 0.01 ^g^	14.33 ± 0.09 ^c^	−7.79 ± 0.05 ^i^	8.49 ± 0.03 ^g^
12	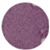	55.05 ± 0.07 ^j^	16.29 ± 0.05 ^b^	−8.63 ± 0.05 ^j^	1.24 ± 0.08 ^k^
13	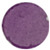	53.50 ± 0.04 ^k^	17.06 ± 0.04 ^a^	−12.61 ± 0.04 ^k^	3.68 ± 0.05 ^j^

^a–k^ For each samples, means with different lowercase letters in the same column are significantly different (*p* < 0.05).

**Table 5:** Comparison between existing labels for response to TVB-N.

Labels	Indicators	Response Mass (g)	Label Area (cm^2^)	Mass/Area (g/cm^2^)	Reference
Carboxymethyl cellulose/pomegranate peel anthocyanins (CMC/PPA)	Pomegranate peel anthocyanins	25	4	6.25	[38]
Cellulose/alizarin composite films (CAF)	Alizarin	10	1.2	8.33	[39]
Pectin/carboxymethyl cellulose sodium/anthocyanins/Zn^2+^ (PC/CMC/ACNs/Zn^2+^)	Anthocyanin	~25	4	~6.25	[40]
Polysaccharide chitosan-anthocyanin-cellulose nanocrystals (CS-AN-CNC)	Blueberry anthocyanin	10	0.25	40	[41]
Cellulose acetate/cobalt-based metal-organic framework (CA/Co-MOF)	Co^2+^	13 ± 2	15	~0.87	[42]
Bacterial cellulose nanofibers/konjac glucomannan-based intelligent film (BCN/KGM)	Curcumin	10	28.26	0.35	[43]
ARS@BRSF-xNaOH-Q		400	0.28	1428.57	This work

## Data Availability

The original contributions presented in this study are included in the article/Supplementary Materials. Further inquiries can be directed to the corresponding author.

## Supplementary Materials

The following supporting information can be downloaded at: https://www.mdpi.com/article/10.3390/foods14162914/s1. Figure S1: N1s spectrum of BRSF-5NaOH-Q (a), BRSF-10NaOH-Q (b), BRSF-15NaOH-Q (c), and BRSF-20NaOH-Q (d).
