# Supplementary figures and images for: A Dual-Functional Intelligent Felt-like Label from Cationic Rice Straw Fibers Loaded with Alizarin Red S for Monitoring Al(III) and the Freshness of Fish
Source: Foods. 2025 Aug 21;14(16):2914. doi: 10.3390/foods14162914 (PMC12385187; doi:10.3390/foods14162914)

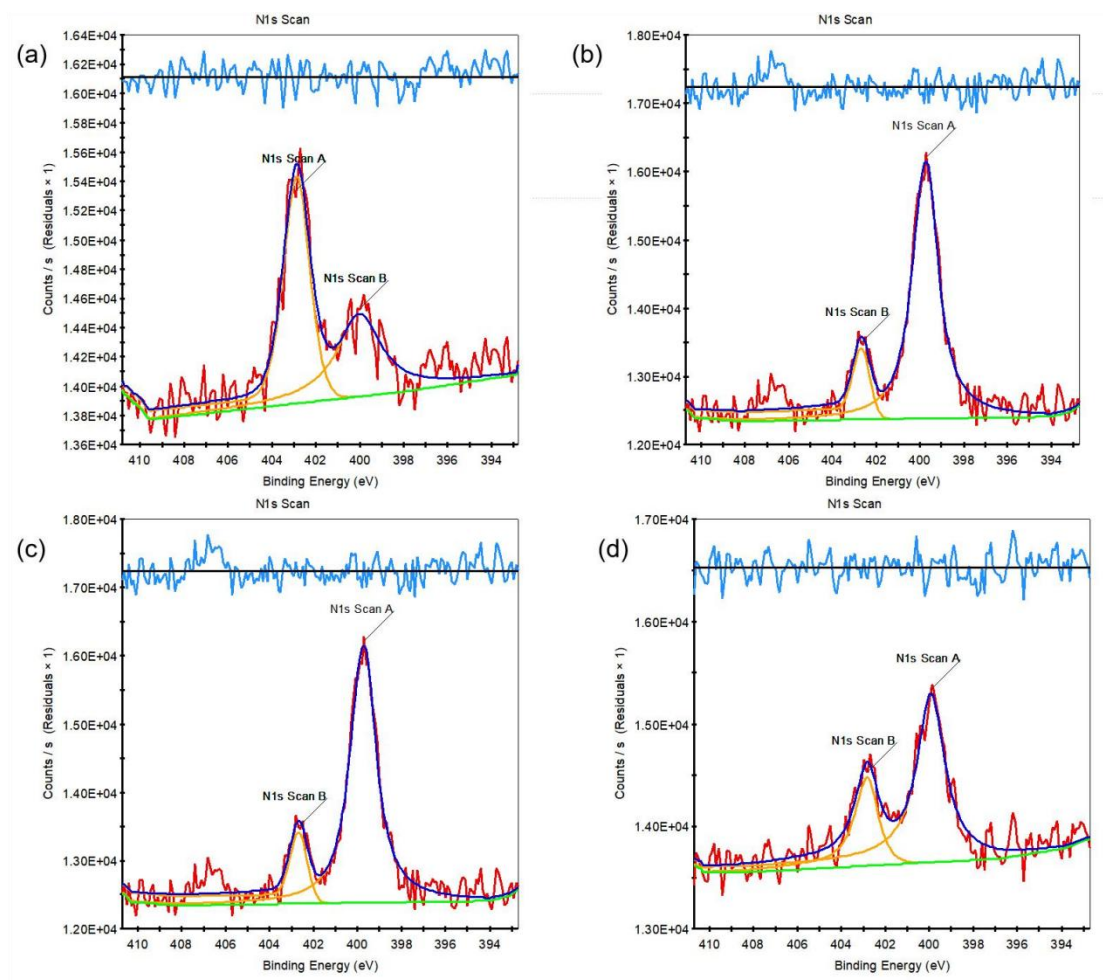

Figure S1. N1s spectrum of BRSF-5NaOH-Q (a), BRSF-10NaOH-Q (b), BRSF-15NaOH-Q (c) and BRSF-20NaOH-Q (d).

Supplement: Supplementary file 1 [file foods-14-02914-s001.zip › foods-3808099-supplementary.pdf]
